# Selected Biomarkers of Tick-Borne Encephalitis: A Review

**DOI:** 10.3390/ijms221910615

**Published:** 2021-09-30

**Authors:** Monika Gudowska-Sawczuk, Barbara Mroczko

**Affiliations:** 1 Department of Biochemical Diagnostics, Medical University of Bialystok, ul. Waszyngtona 15A, 15-269 Bialystok, Poland; mroczko@umb.edu.pl; 2 Department of Neurodegeneration Diagnostics, Medical University of Bialystok, ul. Waszyngtona 15A, 15-269 Bialystok, Poland

**Keywords:** biomarker, tick-borne encephalitis, inflammation, immunoglobulin, free light chain, metalloproteinase, cytokine, chemokine

## Abstract

Tick-borne encephalitis (TBE) is an acute disease caused by the tick-borne encephalitis virus. Due to the viral nature of the condition, there is no effective causal treatment for full-blown disease. Current and nonspecific TBE treatments only relieve symptoms. Unfortunately, the first phase of TBE is characterized by flu-like symptoms, making diagnosis difficult during this period. The second phase is referred to as the neurological phase as it involves structures in the central nervous system—most commonly the meninges and, in more severe cases, the brain and the spinal cord. Therefore, it is important that early markers of TBE that will guide clinical decision-making and the choice of treatment are established. In this review, we performed an extensive search of literature reports relevant to biomarkers associated with TBE using the MEDLINE/PubMed database. We observed that apart from routinely determined specific immunoglobulins, free light chains may also be useful in the evaluation of intrathecal synthesis in the central nervous system (CNS) during TBEV infection. Moreover, selected metalloproteinases, chemokines, or cytokines appear to play an important role in the pathogenesis of TBE as a consequence of inflammatory reactions and recruitment of white blood cells into the CNS. Furthermore, we reported promising findings on tau protein or Toll-like receptors. It was also observed that some people may be predisposed to TBE. Therefore, to understand the role of selected tick-borne encephalitis biomarkers, we categorized these factors and discussed their potential application in the diagnosis, prognosis, monitoring, or management of TBE.

## 1. Introduction

Tick-borne diseases are disorders caused by infection transmitted to humans by tick bites. Tick-borne encephalitis (TBE) is a zoonosis caused by the tick-borne encephalitis virus (TBEV), which infects the human central nervous system. The TBEV is a small RNA virus that belongs to the family of Flaviviridae. This member of the genus Flavivirus was first isolated in 1937 in the USRR [1,2]. In one-third of cases, TBE may cause mild to severe sequelae, including long-term neurological complications or even death. In forested regions of Northern Europe and Asia, an average of 10,000–15,000 cases have been reported annually in recent decades [1,3,4,5]. The clinical spectrum of TBE includes meningitis in around half of patients, and encephalitis, meningoencephalitis, myelitis, or spinal paralysis in others [3]. Patients with TBEV infection may have no symptoms or have symptoms that are nonspecific. Moreover, symptoms may appear from 7 to 14 days after exposure to the virus and, therefore, the diagnosis of TBE can be difficult [6,7]. Two phases of infection in patients with TBE have been observed. The first nonspecific phase manifests without any symptoms or with flu-like symptoms such as fever, headache, or myalgia. TBE may take also a more severe course and, after a first interval, it may proceed to the second phase, which is defined as the neurological phase. The second phase is characterized by fever reaching 40 °C, severe headaches, nausea, vomiting, myalgia, and, finally, meningeal symptoms [3,8,9]. 

The course of TBE varies depending on the subtype of the virus. There are three virus subtypes: European, Siberian, and Far Eastern, the last of which is associated with the highest risk of neurological sequelae and mortality [1]. However, there are measures that can be taken to prevent TBE virus infection. These include the use of insect repellents and protective clothing, and vaccination against TBE, which is offered to individuals in endemic regions. It has been suggested that vaccines based on the European subtype provide protection against the Siberian and Far Eastern subtypes of the virus [10,11].

Disease diagnosis and treatment of patients are based on laboratory test results. However, TBE can be difficult to diagnose due to nonspecific test results in the first phase of the disease, e.g., leukopenia, thrombocytopenia, or liver function tests [12]. A few days after the onset of TBEV infection and appearance of symptoms, specific diagnosis is made by the detection of TBEV-specific IgM and IgG antibodies in the patient’s serum and cerebrospinal fluid [13]. The European Centre for Disease Control (ECDC) has established diagnostic criteria for tick-borne encephalitis. 

According to the ECDC, the disease can be confirmed if two criteria are met: clinical symptoms of central nervous system (CNS) inflammation and at least one of the laboratory confirmation criteria. Laboratory confirmation criteria include: the presence of TBE-specific IgM and IgG antibodies in serum, and/or the presence of TBE-specific IgM or IgM and IgG antibodies in cerebrospinal fluid (CSF), and/or seroconversion or a significant increase in TBE-specific antibodies titer in two serum samples, and/or the detection of TBE viral nucleic acid in CSF, blood, or other body fluid or tissue, and/or isolation of the TBEV from specimen [14,15]. It should be indicated that the virus can be detected by reverse transcriptase–polymerase chain reaction only in the acute phase of the disease. However, patients are most commonly admitted to hospital at the onset of neurological symptoms, when TBE viral nucleic acid is no longer present in blood or CSF [16].

There are also some biomarkers that support the diagnosis of TBE. According to current knowledge, CSF testing helps diagnose conditions affecting the CNS. In the CSF of patients with TBE, moderate pleocytosis with an increased count of segmented granulocytes and increased concentration of protein can be observed [17]. In addition, C-reactive protein or procalcitonin is measured for the differential diagnosis of TBEV infection with other neuroinflammatory disorders caused by bacteria. It has been observed that the levels of abovementioned proteins are most often lower in TBE in comparison to bacterial infections of the CNS [18,19]. However, it must be noted that already used additional biomarkers only support, but do not confirm, the diagnosis of TBE. 

Recent research indicates that the course of TBE is associated with the immune response to the TBEV. There are a number of studies that have explored novel markers of TBE in the available literature. Therefore, the aim of the present review was to present the significance of various inflammatory factors in the diagnosis and prognosis of tick-borne encephalitis.

## 2. Methods

### Literature Search and Data Extraction

**Figure 1:**
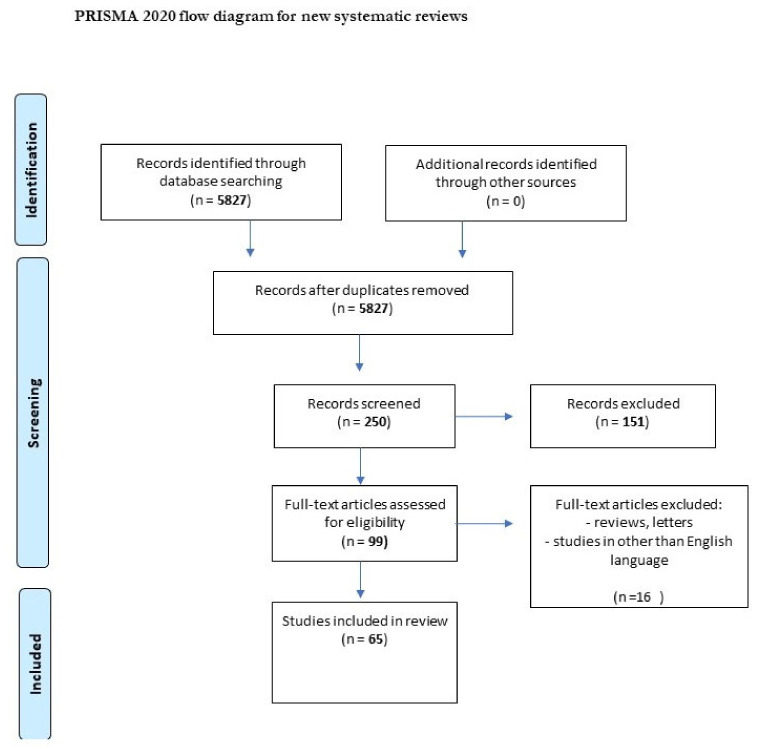
PRISMA flow diagram.

We performed a comprehensive literature search covering the period up to 31 July, 2021. First, we searched the MEDLINE/PubMed electronic database using the keywords: “tick-borne encephalitis” (n = 5827). Then, we used the keywords “chemokine AND tick-borne encephalitis” (n = 16), “metalloproteinase AND tick-borne encephalitis” (n = 9), “gene AND tick-borne encephalitis” (n = 123), and “marker AND tick-borne encephalitis virus” (n = 29). The next step involved limiting the search to studies written in English and the exclusion of duplicates. Thus, 58 publications were included in the study (Figure 1, PRISMA flow diagram modified from Page et al. [20]).

## 3. Results

**Figure 2:**
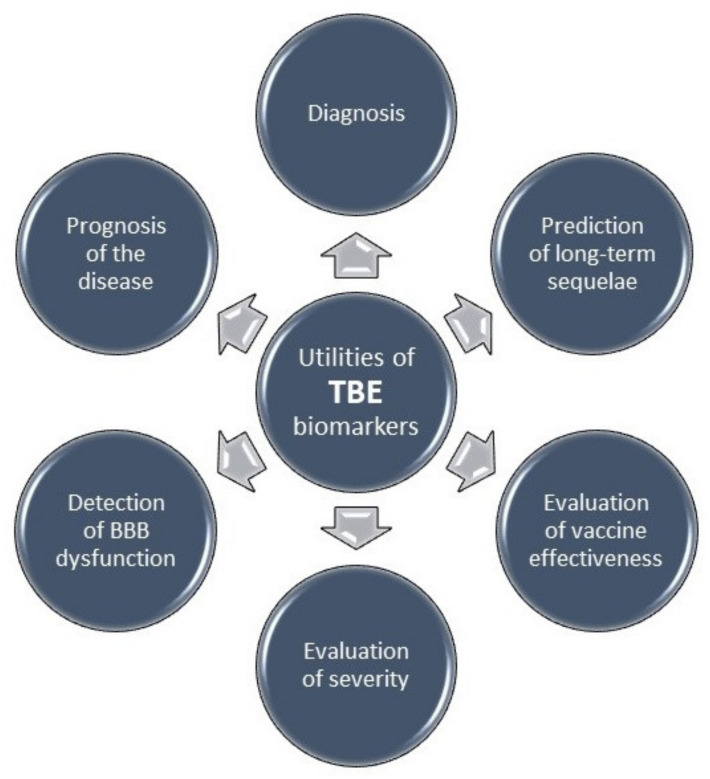
Utilities of TBE biomarkers. TBE, tick-borne encephalitis; BBB, blood–brain barrier.

The role and application of selected tick-borne encephalitis biomarkers are presented in Table 1 and Figure 2.

### 3.1. Specific Immunoglobulins

The routine diagnosis of TBE is based on the determination of specific antibodies against the virus. It is well known that immunoglobulins are protein molecules of the immune system. Specific antibodies are produced by active forms of B lymphocytes in response to an antigen, i.e., a pathogenic viral factor stimulating the immunological system [43]. The serological diagnosis of TBE is based on the determination of IgM antibodies specific to TBEV antigens, the etiological factor of TBE. The specimens of choice for the diagnosis of TBE are blood and CSF. IgM antibodies, whose concentration reaches a peak within a short time of symptom occurrence and persists for around six weeks, are detected following the onset of the second, neurological phase of the infection. Therefore, on admission to hospital, specific IgM antibodies are present in the sera of most patients. The determination of IgG antibody levels is a method for confirming the presence of antibodies following infection or vaccination. Maximum IgG concentrations are detected in late convalescent-phase samples, peak within 3–7 weeks of symptom onset, and persist for a number of years [21,22]. However, it has been reported that TBEV-specific IgG antibodies are present in the sera of 95% of hospitalized patients during the first days of the neurological phase of the disease. On the other hand, 5% of patients that are seronegative have more severe illness, i.e., seronegativity is approximately 20 times higher in patients with meningoencephalomyelitis in comparison to those with meningitis. Moreover, it is associated with the presence of postencephalitic syndrome following infection [44]. 

### 3.2. Free Light Chains (FLCs)

The immunoglobulin molecule consists of heavy and light chains, which are linked by disulfide bridges. There are five types of heavy chains (γ, δ, α, μ, and ε) and two types of light chains (κ and λ). Physiologically, κ and λ light chains are produced in excess in relation to heavy chains, and the synthesis of light chains is around 40% higher in comparison to heavy chains. Light chains that are not combined with heavy chains are released by plasmocytes into the blood as free light chains (FLCs). As a result of filtration in the renal glomeruli, FLCs enter the proximal tubules where they are reabsorbed and metabolized. Most commonly, κ light chains have a monomeric form and a half-life of around 2–3 h, whereas λ light chains are often dimeric and have a longer half-life of up to 6 h. Moreover, the production of κ light chains is approximately twice as high as that of λ light chains. However, κ light chains are cleared around three times faster than λ light chains are [43,45,46,47]. 

It has been observed that immune system stimulation and abnormalities are associated with the abnormal concentration of FLCs [43,45,46,47]. Our previous study demonstrated that pre-treatment concentrations of λ FLCs in the sera of patients with tick-borne encephalitis were significantly higher than post-treatment levels. On the other hand, the concentration of λ FLCs in the CSF was lower in pre-treatment samples than in post-treatment ones. In addition, CSF λ FLCs correlated with CSF TBEV-specific IgM and IgG antibodies. Therefore, increased levels of FLCs are probably caused by the enhanced synthesis of specific immunoglobulins against the TBE virus. Additionally, knowing that the main source of proteins present in the CSF is serum, it has been speculated that increased concentrations of λ FLCs in the CSF result from increased permeability of the blood–brain barrier (BBB). The presence of TBEV-specific antibodies, as well as FLCs, in the CSF may also be an argument for the intrathecal synthesis of immunoglobulins in the CNS during TBEV infection [23].

### 3.3. Matrix Metalloproteinases (MMPs)

Matrix metalloproteinases are endoproteases of the zinc-dependent family. All metalloproteinases are capable of destroying various types of extracellular matrix proteins (ECM), including collagen, proteoglycans, fibronectin, and laminin. MMPs can also affect cell migration, proliferation, and differentiation or contribute to apoptosis, the formation of new vessels, tissue repair, and immune response. They are also able to regulate signaling pathways and are involved in the release of growth factors, growth factor binding proteins, and cytokines [48,49,50]. It has been proven that changes in the activity of metalloproteinases occur in normal physiological processes in the human body, e.g., pregnancy or wound healing [51,52]. However, elevated levels of MMPs are commonly observed in pathological processes, including cardiovascular diseases such as atherosclerosis, diseases of the musculoskeletal system, and cancer [53,54,55,56]. Moreover, studies have indicated that matrix metalloproteinases may be associated with tick-borne encephalitis [26,57,58,59].

MMP-9 is the most widely studied metalloproteinase in TBE. Palus et al. [24] observed that patients with TBE have markedly elevated serum levels of MMPs. This clinical study revealed that males with TBE have higher concentrations in comparison to females, but the concentrations are not age-dependent. It is known that the production of MMP-9 occurs in, inter alia, endothelial cells and that the inhibition of metalloproteinases may reduce brain capillary damage in encephalomyelitis. Therefore, MMP-9 probably plays a pathophysiological role in BBB disruption, and it has been indicated that increased levels of MMP-9 are associated with the increased permeability of the BBB [24]. Similarly, CSF samples have been examined to determine if MMP-9 levels are associated with brain inflammatory response in TBEV infection. In patients in whom the presence of TBEV-specific IgG antibodies was detected, MMP-9 concentrations were higher than in patients who were negative for IgG antibodies. MMP-9 expression was also elevated in all TBE patients who died. Moreover, the correlation between MMP-9 and pro-inflammatory cytokine IL-6 suggests that MMP-9 elevation is related to the inflammatory reaction in the brains of TBE patients [25]. The potential association between TBE and cytokines is described in detail in a later section of this review. 

Interestingly, the MMP-9 gene has been studied to determine the association between MMP-9 genes and a genetic susceptibility to TBE. Barakesh et al. [58] observed that there is a significant link between the rs17576 MMP-9 gene and a severe course of TBE. Moreover, it was reported that the frequency of rs17576 is almost twice as high in patients with meningoencephalitis compared to meningitis [58]. 

The activity and inhibition of MMPs is dependent on tissue inhibitors of metalloproteinases (TIMPs). The most pro-binding inhibitor of MMP-9 is TIMP-1. There is evidence that there are no differences in serum TIMP-1 between TBE patients and controls. However, the MMP-9-to-TIMP-1 ratio is elevated in patients with TBE. This indicates an imbalance between MMP-9 and its most common inhibitor, which may be the reason for the easier entry of the TBEV into the CNS due to BBB disruption [24]. 

Various disorders of the CNS are also associated with A disintegrin and metalloproteinases (ADAMs). ADAMs, originally named metallo/disintegrin/cysteine-rich (MDC) proteins, belong to the Metzincins superfamily of metalloproteases [59]. The role of ADAMs in TBEV infection has been investigated. It has been observed that nine out of 20 ADAMs are overexpressed in cells infected by the TBE virus. Yang et al. [26] revealed that the knockdown of only ADAM15 is associated with a reduction in TBEV RNA levels and nonstructural glycoprotein, NS1. Moreover, in cells with no ADAM15 compared to control cells, TBEV infection is alleviated. On the other hand, TBEV infection is associated with ADAM 15 relocalization from lipid rafts to the endoplasmic reticulum or Golgi regions. Moreover, it has been observed that there is a link between the cellular membrane rearrangements and TBEV replication by the formation of the membranous virus replication organelle. Therefore, these results suggest that ADAM15 probably supports TBEV infection and facilitates virus RNA replication [26]. 

### 3.4. Chemokines and Cytokines

Chemokines or chemotactic cytokines are a large group of proteins that play a vital role in stimulating leukocyte movement and controlling their migration from blood to tissues [60]. Chemokines are polypeptides that have a highly conserved tertiary structure, stabilized by disulfide bonds between cysteines [61]. Considering that most inflammatory chemoattractants are induced and released during acute infection, it has been suggested that there is an association between chemokine levels and TBE. 

Chemokines are produced by, inter alia, neutrophils and it has been demonstrated that neutrophils play an important role in the pathogenesis of TBE. Grygorczuk et al. [27] reported that neutrophils were detected in the CSF of almost 100% of patients on admission to hospital. Additionally, patients with meningoencephalomyelitis (MEM) had a significantly increased neutrophil count in comparison to those with meningitis and meningoencephalitis. On the other hand, at a follow-up appointment two weeks later, pleocytosis was reduced in MEM and the neutrophil count in the CSF normalized faster than did other parameters, such as total protein and albumin concentration. Therefore, the authors examined the concentration of CXCR2 ligands: CXCL1 and CXCL2. Similarly to neutrophils, levels of the chemokines in the CSF were increased on admission. Interestingly, CXCL1 levels were markedly elevated in the CSF compared to serum and decreased two weeks after admission to hospital. The CXCL2 CSF/serum quotient was elevated (1.62) and the CXCL2 concentration remained increased in the second test. These results confirm the intrathecal synthesis of the aforementioned chemokines and indicate their potential role in neutrophile infiltration into the CNS. The roles of CXCL1 and CXCL2 are probably different as only CXCL1 correlates with a lymphocyte count and BBB integrity. Therefore, CXCL1 may facilitate TBEV invasion into the CNS by infected white blood cells [27]. 

It has been observed that CXCR3 expression is also highly increased on human-activated T helper cells type 1 (Th1) during inflammation or tissue damage. The elevation of CXCR3 is mainly due to the increase in interferon-γ (IFN-γ) production and CXCR3 is activated by three IFN-γ-inducible ligands: CXCL9, CXCL10, and CXCL11. As is known, CXCR3 provides the recruitment of Th1 cells into sites of inflammation and it has been suggested that CXCR3 facilitates the interaction between T cells with antigen-presenting cells [62,63,64,65]. Therefore, knowing that the Th1 cell count is elevated at sites of inflammation and that CXCL9 is a ligand for CXCR3, its concentration was examined in TBE patients. There were no significant differences in serum and CSF concentrations of CXCL9 between samples collected in the acute phase of the disease and at a follow-up two weeks after hospital discharge. However, serum and CSF CXCL9 levels in both samples collected from TBE patients were elevated in comparison to controls without inflammation. Moreover, the CXCL9 concentration was elevated in CSF compared to serum, but the differences were significant only in samples collected in the first, acute phase of the disease. The CSF/serum quotient decreased following recovery. Therefore, taking the above into consideration, it appears that CXCL9 participates in the migration of Th1 lymphocytes to the CNS and that it may be involved in TBE immunopathology [28]. Moreover, TBEV infection leads to the extensive production of another CXCR3 ligand—CXCL10 [29,30]. It has been suggested that this proinflammatory factor is one of the first upregulated chemokines in the serum of patients with TBEV infection. Formanova et al. [29] examined changes in the levels of chemokines/cytokines and growth factors in TBEV-infected mice and in human neural cells. The authors observed that TBEV-infected mice demonstrated the time-dependent elevation of, inter alia, CXCL10 in serum samples and brain tissue. The study revealed that the levels of CXCL10 and granulocyte colony-stimulating factor are the first to increase in the serum of infected mice during the viremic phase. The serum concentration of CXCL10 increased rapidly before the virus reached the brain. Therefore, elevated levels of CXCL10 may reflect the viral infection of peripheral tissues. Enhanced CXCL10 levels have also been observed in brain tissue at the time of TBEV invasion into the brain. The levels increased substantially as the infection progressed. Moreover, in line with other studies, the authors observed markedly elevated concentrations of CXCL10 in the CSF of patients with TBEV infection. It seems that CXCL10 plays a role in neuroinflammation via the recruitment of T cells into the CSF and therefore may be a good marker of TBE [29,30,31].

One of the main chemokines recruiting B-cells to the site of infection is also CXCL13. It has been observed that in meningoencephalitis, the concentration of CXCL13 in the CSF, as well as pleocytosis, and the CSF/serum ratio of specific anti-TBEV antibodies were higher in comparison to meningitis. Moreover, following the resolution of symptoms, CXCL13 levels decreased only in patients with meningoencephalitis. It has been revealed that CXCL13 demonstrates a lower but still high performance in discriminating between meningitis and meningoencephalitis in comparison to the total lymphocyte and total cell count in the CFS. Furthermore, correlations between the lymphocyte count, total protein, and albumin concentration in the CSF and CXCL13 suggest its involvement in the movement of lymphocytes expressing CXCR5 from serum to the cerebrospinal fluid [32]. 

Pilz et al. [33] compared CXCL13 concentrations between patients with TBE and those with bacterial or other viral infections of the CNS. The authors demonstrated that among patients with a complicated infection course (bacterial and viral), 50% at admission and 100% at follow-up had markedly elevated CXCL13 levels. Additionally, among patients with an uncomplicated disease course, only those with the Varicella zoster virus had CXCL13 values above the cut-off point (250 pg/mL). Moreover, it was revealed that the determination of CXCL13 is a very good tool for discriminating diseases (AUC = 0.958). The results suggest that the CXCL13 concentration increases with disease progression and development of complications (e.g., encephalitis). Hence, it may indicate the potential role of CXCL13 in the recruitment of B-cells from blood to the CSF. In addition, the albumin quotient (Q Alb = Albumin in CSF/Albumin in serum), an indicator of BBB permeability, differed significantly between patients with different disease courses. Knowing that Q Alb is an indicator of BBB damage and CXCL13 across impaired blood–brain barriers from blood, we speculate that the degree of CXCL13 diffusion during neuroinfection may be associated with the value of the albumin quotient [33].

Several monocyte-attracting chemokines have also been measured in the blood and CSF of TBE patients. The concentrations of three of them, CCL2, CCL7 and CXCL12, as well as the concentration of platelet-endothelial cell adhesion molecule-1 in the CSF, were found to be elevated in TBE. However, only CCL7 levels were shown to be higher in TBE than in non-TBE meningitis. Moreover, it was indicated that the monocyte count in the CSF correlates with other inflammatory markers. Based on these results, increased intrathecal synthesis could be associated with elevated levels of chemokines CCL2, CCL7, and CXCL12, which results from an increased requirement for monocytes, platelets, and T-cells in the CNS [34,35].

A different study evaluated levels of chemokines together with cytokines, 24 proteins in total associated with immune response (B cell: CXCL12, CXCL13; Th1: IFNγ, IL-12P40, IL-12P70, CXCL10, CXCL9, CCL19; Th17: IL-17F, IL-17A, IL-22, IL-21, IL-23, IL-25, IL-27; innate: GMCSF, IFNα, IL-1β, IL-6, IL-8, TNFα, CCL2, CCL3, IL-10). Bogovič et al. [66] performed analyses at three time points: on admission, 2 months, and 2–7 years following hospital discharge. Levels of the investigated chemokines/cytokines were similar in the acute phase of the disease and 2 months after recovery. However, it was observed that at the final appointment, levels of Th17 mediators were decreased in patients with post-encephalitic syndrome (PES), whereas levels of Th1 mediators or B-cells were elevated in this group of patients. Therefore, changes in these mediators are probably associated with pathogenesis and an inflammatory response persisting for years after TBEV infection [66]. 

Both pro- and anti-inflammatory cytokines present in the CSF are involved in the immune response against the TBEV. In children and adults with TBE, the concentration of one of the most important proinflammatory markers, IL-6, is considerably increased. The level of IL-6 in the acute phase of this viral disease is higher in comparison to neuroborreliosis (NB) caused by a bacterium called Borrelia burgdorferi or healthy subjects. By contrast, levels of IL-7, -8, -13, and -10 are lower in TBE in comparison to NB in the acute phase of the diseases and before treatment [25,36,39]. Moreover, knowing that IL-10 is a very powerful anti-inflammatory cytokine, the ratio of IL-6 to IL-10 was calculated. It was observed that the ratio was higher in TBE than in other groups mentioned above, indicating an imbalance between pro- and anti-inflammatory factors in favor of IL-6. This may be the reason for long-lasing and permanent sequalae frequently experienced by TBE patients [61]. These results may suggest that the etiology of neuroinflammation is related to probably different neuroinflammatory signaling pathways [25,36,39]. Similarly, it has been revealed that the concentration of IL-5, which is responsible, inter alia, for the proliferation and production of antibodies, is elevated in the CSF of TBE patients. Moreover, an elevated CSF/serum IL-5 index indicates its synthesis in the CSF or increased permeability of the BBB. However, it was surprising that its concentration did not correlate with the clinical course of the disease or specific anti-TBEV IgM and IgG titers. Therefore, the exact role of IL-5 in the pathogenesis of TBE is still unclear [38].

### 3.5. Genetic Factors

To date, a genetic predisposition to TBE has been poorly examined, but it has been suggested that some host genetic factors may have an impact on the severity of TBE. Kindberg et al. [67] hypothesized that CCR5, a chemokine receptor for CCL4, CCL5, CCL8, CCL11, CCL13, and CCL16, may affect the host immune response in TBEV infection. Patients with TBE were investigated for a 32-base-pair deletion in the CCR5 gene. The scientists performed genotyping in TBE patients, patients without TBE-specific antibodies but with CSF pleocytosis suggesting a viral etiology of aseptic meningoencephalitis (AME), and in the control group. They observed that patients with aseptic meningoencephalitis and the controls had similar allele distributions. Homozygotes with CCR5Δ32 were only patients with TBE, and the difference between TBE patients and other groups was significant. Similarly, CCR5Δ32 prevalence was increased in TBE in comparison to AME patients [67]. CCR5 mutation was also found to be higher in children than in TBEV-naive and AME controls [68]. Additionally, the allele prevalence correlated with disease severity. Therefore, it would appear that homozygotes, as well as heterozygotes with the CCR5 mutation, are more susceptible to TBEV infection [67,68]. 

Barkhash et al. [37] evaluated the role of single nucleotide polymorphisms of rs3109675 in the COL5A1 gene, intronic rs41554313 in the POLRMT gene, and intergenic rs10006630 in TBE susceptibility. It was found that rs10006630 located on chromosome 4 is associated with an enhanced predisposition to TBE and increased susceptibility to a severe disease course [37]. Moreover, it has been observed that there is an association between genes located on chromosome 7 (Cd33, Klk1b22, Siglece, Klk1b16, Fut2, Grwd1, Abcc6, Otog, and Mkrn3) and patient survival following TBEV infection. The authors described that, e.g., the Cd33 gene probably regulates the immune response, whereas Klk1b16 activates the complement system and may cause a worsening of inflammation symptoms. Moreover, it has been suggested that some of the genes, e.g., Fut2 or Mkrn3, are associated with the defense against viruses. Additionally, it has been described that all potential candidate genes are expressed, inter alia, in the brain, and it is well known that the TBEV directly infects the brain and causes encephalitis. However, further research on human samples is needed to evaluate the association between genes located on chromosome 7 and the susceptibility to TBEV infection [69]. 

It has been indicated that another factor affecting the course of TBE is a different component of the innate immune system—Toll-like receptor 3 (TLR3). TLR3 plays a crucial role in virus recognition, and, therefore, Mickiene et al. [68] examined the polymorphism of TLR3. Interestingly, the homozygous TLR3 rs3775291 genotype was less prevalent among children and adults with TBE than among control subjects. Moreover, in the total study group, the genotype and allele prevalence of TLR3 did not differ significantly depending on disease severity, and the homozygous mutant TLR3 rs 3775291 genotype did not correlate with the severity of TBE. However, the homozygous TLR3 genotype was less prevalent in adults with a severe disease course compared to total adult TBEV infection cases. Hence, it can be presumed that the homozygous functional rs3775291TLR3 genotype is associated with the clinical course of TBE in adults, but the TLR3 polymorphism is probably not a risk factor of TBE in children [68].

Considering the above, it may be speculated that the genetic modulation of the immunological system may probably provide an option for the management of TBE. However, there is a need for further studies to confirm that assumption.

### 3.6. Other Biomarkers

Interestingly, it has been suggested that a bioactive lipid mediator, sphingosine-1-phosphate (S1P), is associated with inflammation in TBE. It has been observed that S1P levels are elevated in TBE and decrease after treatment in most severe cases. S1P induces the production of previously mentioned proinflammatory cytokines, including IL-6. Therefore, this may be evidence of the impact of S1P on the inflammatory response to tick-bites, and it may provide a potential basis for finding new specific treatments against the TBEV in the future [39].

Another marker associated with TBE is high electrophoretic mobility group B1 protein (HMGB-1), which is considered to be a DNA binding molecule. HMGB-1 is released from cells into the extracellular environment and acts on specific cell-membrane receptors. It has been demonstrated that HMGB-1 is involved in chemotaxis or immune system modulation [40,70]. Moniuszko-Malinowska et al. [40] compared CSF and serum HMGB-1 concentrations in TBE and NB patients. The concentration of HMGB-1 was associated with both viral and bacterial infection. Serum levels of HMGB-1 were not elevated in TBE in comparison to healthy individuals, whereas CSF concentrations were higher in TBE than in controls. Moreover, differences in serum HMGB-1 levels between TBE and NB were statistically significant. The HMGB-1 serum concentration was significantly higher in samples obtained from patients with NB in comparison to TBE [40]. 

Another potential biomarker of TBE is Toll-like receptor-2 (TLR-2). TLR-2 plays different roles during inflammation, i.e., it stimulates the activation of antigen-presenting cells and the production of cytokines or modulates the immunological system. In contrast to HMGB-1, serum TLR-2 levels were found to be significantly increased in TBE in comparison to controls. Other TLR-2 associations in TBE and NB were similar to HMGB-1. Summarizing, serum HMGB-1 and TLR-2 concentrations appear to be useful early diagnostic biomarkers of TBE and good tools for differentiating TBE from NB [41].

Tau protein concentrations in TBE patients and healthy subjects were analyzed. Patients with TBEV infection were divided into two groups: patients with meningitis and patients with meningoencephalitis. It has been observed that 14 days after treatment, the tau concentration was significantly higher in patients with meningoencephalitis in comparison to patients without inflammation in the CNS. Moreover, the tau protein concentration in all patients with TBE was higher after therapy in comparison to samples taken before treatment. There was also a difference between patients with sequalae and without sequalae—patients with sequalae had higher tau concentrations in comparison to those without. It seems that the analysis of tau protein may reflect CNS damage during TBE infection [42]. Based on current knowledge about the abnormal hyperphosphorylation of tau protein in pathological neurodegeneration, e.g., in Alzheimer’s disease, it is suggested that, in general, patients with markedly elevated tau protein levels are more vulnerable on a sequalae because of inflammation and neurodegeneration processes [42,71]. 

The determination of neuron-specific enolase (NSE) in serum is useful in the diagnosis and therapy of small-cell lung carcinoma and neoplasms originating from the neuroendocrine system. Apart from being found in selected healthy cells, NSE is present mainly in neuroblastoma, gliomas, small-cell neoplasms, or thymoma. During cell necrosis, NSE can be passively released into the bloodstream [72,73]. A marginal increase in serum NSE concentration has also been observed in head injuries or meningitis. In turn, the S100B protein is a calcium-binding molecule belonging to the S100 protein family. It is present in the cytoplasm of the choroid plexus of astrocytes and oligodendrocytes, from which it is actively released. Increased S100B protein levels in serum and the CSF are considered indicators of glial activation [74]. As both NSE and S100B are associated with CNS disorders, changes in their concentrations in TBE patients have been examined. It should be pointed out that NSE values in the CSF were significantly higher in patients with meningoencephalitis in comparison to individuals with meningitis and controls, while S100B levels were similar in TBE and the control group. Moreover, it was observed that post-treatment, serum S100B concentrations were markedly elevated in the sequelae group. This may suggest that neurodegeneration occurs even after recovery and that NSE determination could be used in the prediction of disease course [75]. 

## 4. Conclusions

Tick-borne encephalitis is a viral disease that causes flu-like and, in more severe cases, neurological symptoms. The total number of infected people has not been conclusively determined, as diagnosis in the early first phase characterized by the lack of or nonspecific symptoms is difficult. Routine laboratory diagnosis of TBEV infection is based on a serological examination, but recent studies have suggested that other biomarkers may be useful predictors of TBE. The use of additional biomarkers in the first stages of disease can significantly reduce serious complications and mortality caused by tick-borne encephalitis virus. 

This review gives an overview of the current literature presenting the role and potential usefulness of tick-borne encephalitis biomarkers. Published research results describe that patients with TBE have elevated levels of λ free light chains in the CSF. Knowing that free light chains are produced in excess during the synthesis of immunoglobulins, it was speculated that a markedly elevated concentration of λFLCs in CSF is caused by the production of antibodies against the tick-borne encephalitis virus. On the other hand, the presence of free light chains in CSF may be associated with increased permeability of the blood–brain barrier in patients with TBEV infection. Moreover, it has been observed that the levels of metalloproteinases, cytokines, and chemokines are associated with disease severity, increased invasion of the TBEV into the CNS, or blood–brain barrier disruption. It was presented that, e.g., MMP-9, which may be responsible for the development of the CNS or induction of apoptosis, is associated with increased permeability of the BBB in TBE. In addition, it was speculated that IL-6, which has very important and multiple roles in the immune response, contributes to the severity of the disease and permanent sequalae. Moreover, chemokines were widely studied in terms of potential biomarkers of TBE. It has been speculated that chemokines may facilitate TBEV invasion into the CNS by infected white blood cells due to their role in lymphocyte migration and that the chemokine receptors are expressed by the intrathecal Th lymphocyte. It has also been indicated that increased levels of tumor markers (e.g., NSE) may reflect the damage of neurons during the acute phase of TBE. In addition, the increased concentration of tau protein in the sequelae group may suggest that neurodegeneration caused by TBEV infection can be expressed by an increased tau protein level. In addition, some genetic factors are probably associated with a predisposition to a severe course of TBE. However, it should be pointed out that some of the studies were performed on animal models and, unfortunately, models used in research do not always reflect the human organism. Therefore, a comparison between animals and the human samples will probably help to understand the function of biomarkers associated with TBE.

Summarizing, the presented results suggest the potential application of TBE biomarkers in the diagnosis, prognosis, monitoring, or treatment of TBE. Despite the numerous candidate biomarkers, it seems that, inter alia, chemokines (CXCL1, -2, -9, -12, CCL2, -7), HMGB-1, or TLR-2 may be used as potential and additional biomarkers of TBE. Moreover, changes in the concentrations of λ free light chains, MMP-9, or CXCL1 are probably associated with dysfunction and increased permeability of the BBB. Additionally, a high potential application of CXCL13, CCL7, and HMGB-1 was reported for distinguishing TBE meningitis from other CNS disorders. 

We believe that measurements of the described biomarkers might become useful in supporting the diagnosis of TBE at clinical practice in the future. However, further studies are needed to confirm the findings presented in this review.

## Figures and Tables

**Table 1:** Applications of selected tick-borne encephalitis biomarkers.

Biomarker	Level and Material	Application	References
TBEV-specific IgM and IgG	↑ in serum, CSF	▪ Potential and additional biomarker for diagnosis of TBE ▪ May evaluate the effectiveness of TBE vaccination	[13,21,22]
λ free light chains	↑ in CSF ↓ in serum—after treatment	▪ May detect dysfunction and increased permeability of BBB ▪ Correlate with intrathecal synthesis of TBEV-specific antibodies	[23]
MMP-9	↑ in serum, CSF	▪ May detect BBB dysfunction and increased permeability ▪ Correlates with brain inflammatory response and synthesis of TBEV-specific antibodies	[24,25]
ADAM15	↑ in cells infected with TBEV	▪ May contribute to TBEV infection and virus RNA replication	[26]
CXCL1	↑ in CSF—on admission ↓ in CSF—after treatment	▪ Potential and additional biomarker for diagnosis of TBE ▪ Associated with neutrophils infiltration into CNS ▪ Correlates with lymphocytes count and increased permeability of BBB	[27]
CXCL2	↑ in CSF—on admission and after treatment	▪ Potential and additional biomarker for diagnosis of TBE ▪ Associated with neutrophils infiltration into CNS ▪ May increase TBEV invasion into CNS	[27]
CXCL9	↑ in serum, CSF ↓ CSF-to-serum ratio after recovery	▪ Potential and additional biomarker for diagnosis of TBE ▪ May be involved in TBE immunopathology	[28]
CXCL10	↑ in serum, CSF and brain tissues	▪ Diagnosis of TBE ▪ May reflect viral infection of peripheral tissues and CNS	[29,30,31]
CXCL13	↑ in CSF ↓ in CSF—after recovery	▪ May distinguish meningoencephalitis from meningitis ▪ Involved in lymphocytes movement ▪ Associated with complications	[32,33]
CCL2, CCL7, CXCL12	↑ in CSF	▪ Potential and additional biomarker for diagnosis of TBE ▪ May detect dysfunction and increased permeability of BBB ▪ May distinguish TBE meningitis from non-TBE meningitis (CCL7)	[34,35]
IL-6	↑ in CSF	▪ Associated with acute phase of disease ▪ Associated with sequalae	[25,36,37]
IL-10	↓ in CSF	▪ Decreased level may be associated with severe course of TBE	[36]
IL-5	↑ in CSF	▪ Correlates with intrathecal synthesis and increased permeability of BBB	[38]
S1P	↑ in CSF—on admission ↓ in CSF—after treatment	▪ Correlated with IL-6 synthesis ▪ May have influence on inflammatory response	[39]
HMGB-1	↑ in CSF ↑ in serum (NB > TBE)	▪ Potential and additional biomarker for diagnosis of TBE ▪ Associated with development of inflammation ▪ May distinguish TBE from NB	[40]
TLR-2	↑ in serum	▪ Potential and additional biomarker for diagnosis of TBE	[41]
Tau protein	↑ in CSF—after treatment	▪ May indicate neurodegeneration process ▪ May be used as a predictor of complicated course of TBE	[42]

TBEV, tick-borne encephalitis virus; MMP-9, matrix metalloproteinase 9; ADAM15, disintegrin and metalloproteinase domain-containing protein 15; CXCL, C-x-C motif ligand; CCL, C-C motif ligand; IL, interleukin; S1P, sphingosine-1-phosphate; HMGB-1, high electrophoretic mobility group B1 protein; TLR-2, Toll-like receptor 2; CSF, cerebrospinal fluid; NB, neuroborreliosis; TBE, tick-borne encephalitis; BBB, blood–brain barrier.

## Data Availability

Not applicable.
